# Improving Incident Reporting in a Hospital-Based Radiation Oncology Department: The Impact of a Customized Crew Resource Training and Event Reporting Intervention

**DOI:** 10.7759/cureus.14298

**Published:** 2021-04-05

**Authors:** Susan L Swanson, Sean Cavanaugh, Felipe Patino, John W Swanson, Corrine Abraham, Carolyn Clevenger, Elaine Fisher

**Affiliations:** 1 Patient Safety, Quality Improvement and Systems Leadership, Emory University Nell Hodgson Woodruff School of Nursing, Atlanta, USA; 2 Radiation Oncology, Cancer Treatment Centers of America Southeastern Medical Center, Newnan, USA; 3 Radiation Oncology, Landauer Medical Physics, Sharpsburg, USA; 4 Quality Improvement, Emory University Nell Hodgson Woodruff School of Nursing, Atlanta, USA; 5 Curriculum and Accreditation, Emory University Nell Hodgson Woodruff School of Nursing, Atlanta, USA

**Keywords:** incident learning system, incident, error, near miss, crew resource management, radiation oncology, patient safety, high reliability, joint commission, quality improvement

## Abstract

Background

Radiation oncology (RO) is a high-risk environment with an increased potential for error due to the complex automated and manual interactions between heterogeneous teams and advanced technologies. Errors involving procedural deviations­­ can adversely impact patient morbidity and mortality. Under-reporting of errors is common in healthcare for reasons such as fear of retribution, liability, embarrassment, etc. Incident reporting is a proven tool for learning from errors and, when effectively implemented, can improve quality and safety. Crew resource management (CRM) employs just culture principles with a team-based safety system. The pillars of CRM include mandatory error reporting and structured training to proactively identify, learn from, and mitigate incidents. High-reliability organizations, such as commercial aviation, have achieved exemplary safety performance since adopting CRM strategies.

Objective

Our aim was to double the rate of staff error reporting from baseline rates utilizing CRM strategies during a six-month study period in a hospital-based radiation oncology (RO) department.

Methods

This quasi-experimental study involved a retrospective review of reported radiation oncology incidents between January 2015 and March 2016, which helped inform the development and implementation of a two-step custom CRM training and incident learning system (ILS) intervention in May 2016. A convenience sample of approximately 50 RO staff (Staff) performing over 100 external beam and daily brachytherapy treatments participated in weekly training for six months while continuing to report errors on a hospital-enterprise system. A discipline-specific incident learning system (ILS) customized for the department was added during the last three months of the study, enabling staff to identify, characterize, and report incidents and potential errors. Weekly process control charts used to trend incident reporting rates (total number of reported incidents in a given month /1000 fractions), and custom reports characterizing the potential severity as well as the location of incidents along the treatment path, were reviewed, analyzed, and addressed by an RO multidisciplinary project committee established for this study.

Results

A five-fold increase in the monthly reported number of incidents (n = 9.3) was observed during the six-month intervention period as compared to the 16-month pre-intervention period (n = 1.8). A significant increase (>3 sigma) was observed when the custom reporting system was added during the last three study months.

Conclusion

A discipline-specific electronic ILS enabling the characterization of individual RO incidents combined with routine CRM training is an effective method for increasing staff incident reporting and engagement, leading to a more systematic, team-based mitigation process. These combined strategies allowed for real-time reporting, analysis, and learning that can be used to enhance patient safety, improve teamwork, streamline communication, and advance a culture of safety.

## Introduction

Radiation oncology (RO) is a complex and high-risk environment that has an increased error probability. While advances in RO technology and treatment techniques have contributed to reduced patient morbidity and mortality, automated and manual processes, combined with complex interactions between heterogeneous teams, can result in undetected errors throughout a patient's treatment path. Failures in communication, leadership, and decision-making, in a culture of retribution, are known factors contributing to unintended consequences and adverse outcomes in complex settings such as RO. While the majority of radiotherapy incidents are minor and often preventable, a single error can result in catastrophic harm or significantly affect multiple patients. The New York Times (2010) seminal series on the devastating effects of treatment errors from technology advancements in radiation oncology [1] has heightened industry mitigation efforts and recommendations for comprehensive safety and quality improvement programs that include incident learning. According to industry experts, strategies for mitigation can best be developed only after understanding the nature, potential frequency, and severity of such events through incident learning [2]. Incident learning involves systematically capturing information on errors and near misses (errors that are intercepted before they reach the patient) to improve the quality and safety of radiation oncology treatments [3]. In fact, Clark et al. cite the use of incident reporting as an indirect measure of system safety and a surrogate to indicate changes in a (radiation oncology) safety program [4].

The use of incident learning as a proven quality and safety tool in high-reliability organizations (HROs), such as commercial aviation, is gaining prominence in radiation oncology [5]. HROs proactively focus on the systematic identification and elimination of “latent conditions” and event precursors rather than a reactive analysis of high-profile adverse events [6]. Commercial aviation is recognized for achieving decades of exemplary safety performance since adopting a proactive and comprehensive, risk mitigation program called crew resource management (CRM). The goal of CRM is to minimize the effect of human error and maximize human performance through enhanced communication, situational awareness, decision-making, and teamwork in a sound safety climate. CRM combines mandatory error reporting, a cornerstone for incident learning, with structured team safety training and standardized protocols to optimize “crew” safety performance [7]. The Joint Commission supports team training and an Institute of Medicine (IOM) report specifically recommends the healthcare industry institute team training based upon commercial aviation’s CRM strategies [8].

An approach combining both incident learning and key strategies from CRM may be clinically useful to identify, prevent, and/or mitigate potential incidents and adverse events in patients undergoing radiation oncology treatment. This includes identification of near misses, which in radiation oncology, may include errors detected and immediately corrected when medical physicists perform a "second check" as part of a RO department's quality program. Several studies have reported results from single interventions designed to mitigate errors or improve reporting rates [2-3,5,7], however, improvement research evaluating the impact of dual or multi-intervention programs (i.e., team-based safety combined with a discipline-specific reporting system) continues to evolve [6,9].

This study describes the development, implementation, and impact of a six-month, two-pronged team-training and incident-learning intervention adapted from CRM principles on the rate of incident reporting in a complex radiation oncology setting. Incident severity categories, points of incident origination, and other factors potentially contributing to reporting outcomes were also assessed.

## Materials and methods

Study design

**Figure 1:**
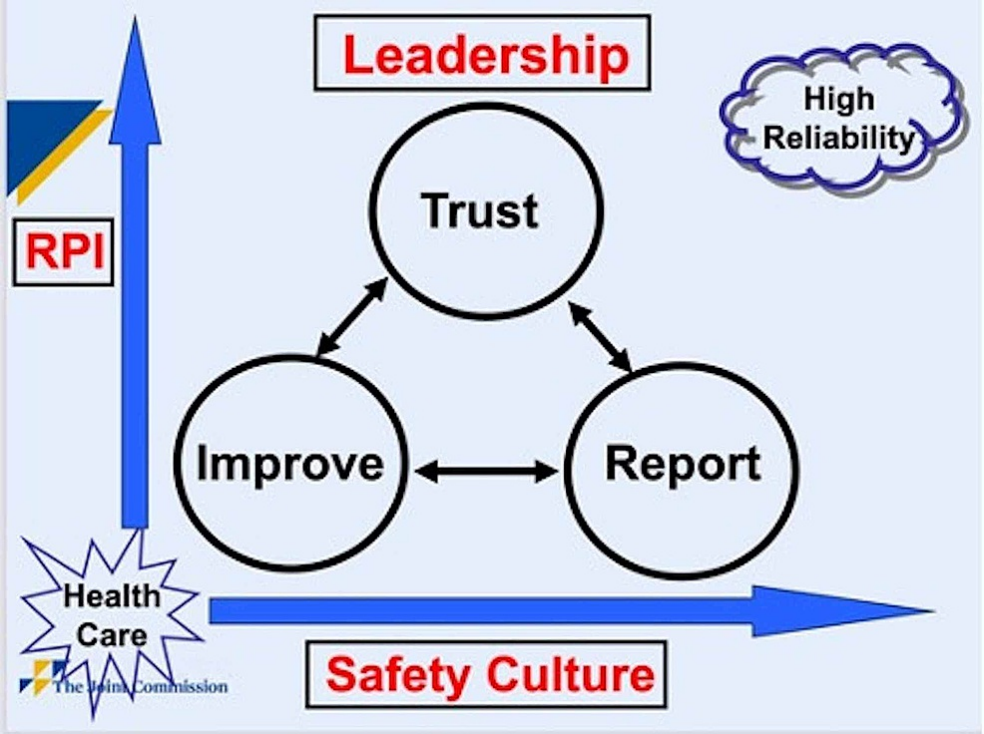
The Joint Commission RPI Model The Joint Commission RPI Model for high-reliability healthcare reflects a commitment to zero harm. Leadership promotes trust through a culture of non-retribution, where staff feel comfortable reporting and learning from incidents, resulting in continuous robust process improvement. (Figure used with permission from the Joint Commission) RPI: Rapid Process Improvement

A two-pronged training and incident reporting intervention, with a convenience sample of over 50 staff participants in a community-based radiation oncology facility, was implemented in May 2016. The High-Reliability Health Care Framework and Rapid Process Improvement (RPI) model (Figure 1) adapted from The Joint Commission Patient Safety Systems Comprehensive Accreditation Manual for Hospitals guided this study in accordance with three designated domains [10]: 1. Leadership's commitment to the goal of "Zero Harm" (the ultimate goal for achieving a high-reliability safety system); 2. An organizational safety culture where all staff feel free to speak up about anything that may negatively impact the organization without fear of retribution; and 3. An empowered workforce that employs Robust Process Improvement (RPI) tools to address any improvement opportunities they find and drive significant and lasting change [11].

Baseline data collected during the 16-month pre-intervention period (Phase I) included a retrospective review of the number of reported radiation oncology incidents (n=29) recorded on the hospital enterprise event reporting system (H-ERS) from January 2015 to April 2016. The H-ERS, managed by the hospital Quality Department, offered no ability to detail or characterize incidents, such as near misses and close calls (errors that reached the patient but did not cause harm), in a discipline-specific manner (i.e. dose, location, severity, etc.). Near-miss and other such incidents were reported verbally or in writing to the medical physics team for follow-up and mitigation, as appropriate.

**Figure 2:**
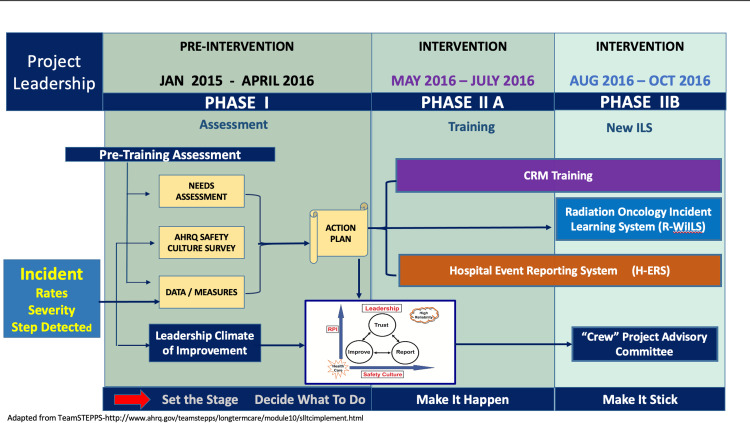
Study Phases The six-month intervention involved two three-month phases. Phase IIA involved department-specific Crew Resource Management (CRM) training during weekly staff huddles while staff continued to report on the hospital event reporting system (H-ERS). Phase IIB added a new radiation oncology web-based incident learning system (R-WILS) and a multidisciplinary project committee to staff weekly training and the concurrent use of the H-ERS.

A 20-question Likert-scale survey on safety culture adapted from the Association of Healthcare Research and Quality (AHRQ) [12] was administered to staff in April 2016 to gauge knowledge, attitudes, and perceptions of the department's safety climate. Survey responses helped inform customization of the CRM training program as well as staff readiness for project participation. Training modules were structured around radiation oncology industry best practices, adverse events, and high-reliability organization principles in addition to informally reported "close calls" within the department. Weekly CRM team training was introduced during the first three months of the study (Phase IIA), followed by the addition of an incident learning system (ILS) specifically created for the department (Figure 2) for the final three-month study period (Phase IIB).

Setting 

The department in this study was one of five national oncology hospital-based radiation oncology (RO) programs sharing an enterprise-wide electronic medical record and generic H-ERS. Each site housed a unique radiation oncology patient treatment database, multi-modality technology configuration, and treatment workflow that were not integrated with the national enterprise electronic health record system (EHR) or the H-ERS. Three of the five departments were supported by a national medical physics group contracted to function as in-house consultants. The remaining two departments utilized employed medical physicists. The contract sites, lead by a chief of medical physics for the group, routinely collaborated and audited one another utilizing enterprise-wide industry standards and best practices between their respective sites. The contract group collaborated with the employed medical physicists as requested and offered access to the group's quality and safety program materials.

The chief of physics for the contract group was based at the department in this study. The department staff, comprising radiation oncology physicians, medical physicists, radiation therapists, nurses, and support personnel, typically treated over 100 patients daily on one of three advanced linear accelerators, an intra-operative treatment unit, and/or in an active brachytherapy program. Individual patient treatment regimens ranged from one session (single fraction) to multiple sessions over a six to eight-week period (30-40 fractions).

Study phases

Phase I - Preintervention

Prior to study implementation, department leadership (administrator, medical director, and chief of medical physics) participated in three structured training sessions aligned with The Joint Commission RPI Model (Figure 1) to prepare for their roles as project mentors and managers. The topics included: 1) structured communication and incident learning strategies, 2) data collection, analysis, and reporting, and 3) role modeling, safety culture, and incentives to hardwire staff safety and reporting behaviors. The leadership assisted in identifying a multidisciplinary Project Committee (Committee) to guide project deliverables, including staff training and incident learning activities. The Committee used baseline incident information including: a) industry recommendations for incident learning systems [3]; b) data from the H-ERS; feedback from team huddles; staff responses from the safety culture survey [12], and c) The Joint Commission RPI Model to help tailor the design of the custom radiation oncology web-based ILS (R-WILS) platform and department CRM training curriculum.

Phase IIA - CRM Training

Fifty CRM training sessions were delivered to staff over a six-month intervention period in two, separate 45-minute, weekly staff huddles while staff continued reporting on the H-ERS (Phases IIA and IIB). Weekly training sessions were intentionally built into the normal departmental workflow to help “hardwire” team-based safety behaviors, increase knowledge and comfort with reporting, provide routine feedback, and minimize staff perceptions of additional workload or a study “halo effect.” Sessions included interactive lectures incorporating video vignettes and slides of industry case studies; methods and tools for incident recognition, characterization, and reporting criteria; team-based safety and risk mitigation strategies, such as leadership, situational awareness and assertiveness; structured communication techniques; and other team-based safety protocols. For example, Team Strategies and Tools to Enhance Performance and Patient Safety (TeamSTEPPS™) [13], a high-reliability safety program created by the Department of Defense, utilizes structured communication in the form of an acronym SBAR (Situation, Background, Analysis, and Recommendation). The Joint Commission [10] strongly endorses SBAR as a powerful and proven tool for effective and accurate communication among healthcare workers. Staff were trained to use SBAR to build greater fidelity and quality in the transfer of critical information within the R-WILS and in other patient or incident-related exchanges. Additional safety tools, such as procedural checklists and timeouts at critical junctures, where incidents were identified as most likely to occur, were also developed but not evaluated in this study. 

Additional high-reliability strategies consistent with the Joint Commission RPI Model [11] were used to promote and sustain desirable staff safety and reporting behaviors. For example, the leadership proactively encouraged reporting and a safety culture by positively reinforcing staff to share any and all events, without fear of retribution. The medical director worked to instill trust and transparency by openly sharing his reported near misses while soliciting input from staff on mitigation strategies and recommendations for improvement. He and the chief of medical physics consistently acknowledged the team for their vigilance when reviewing incidents during huddle CRM training sessions and during Committee meetings. Processes were also put in place for constructive follow-up by leadership that included positive feedback when staff members reported incidents, in addition to publicly acknowledging staff for actionable engagement in both CRM training and incident reporting on the new R-WILS. Aviation “lapel wings” were distributed by the leadership to solidify staff engagement and commitment as members of the radiation oncology “crew” supporting high-reliability healthcare and zero harm. In fact, staff were expected to (and were observed) wearing “their wings” throughout the study in an effort to visually hardwire a team-based safety culture.

Phase IIB - The Radiation Oncology Web-based Incident Learning System (R-WILS)

Because the H-ERS lacked RO-specific incident characterization and was not well-utilized, the department leadership and the author designed the 20-question, in-house, RO-specific, web-based, voluntary electronic incident learning system (R-WILS) to be used concurrently with the H-ERS for this study. While there were a few commercially available incident reporting applications, one proven, national tool called Radiation Oncology Incident Learning System (ROILS), launched by the American Association of Physicists in Medicine [14], provided an industry-specific reference from which we customized the R-WILS format. We selected a web-based commercial survey tool called SurveyGizmo [15] as the platform to support our R-WILS application since this software was intuitive and provided the breadth of survey, analytics, and graphics capabilities for custom reporting. Lean-six sigma methods were used to clarify workflow through process mapping; the potential for events through causal diagrams; and the patient and staff journey using direct observation. Staff input from each functional area helped validate each step of the customization process. This included department-specific attributes, criteria, and the 20-item Likert scale questions for the R-WILS reports with related analysis for a) incident type (clinical or non-clinical), b) incident-related definitions (i.e., near miss, event, error, etc.), c) potential severity categories, d) origination steps, e) reporting rate metrics, and f) an HRO-acronym (SBAR) for standardized communication of an occurrence.

**Figure 3:**
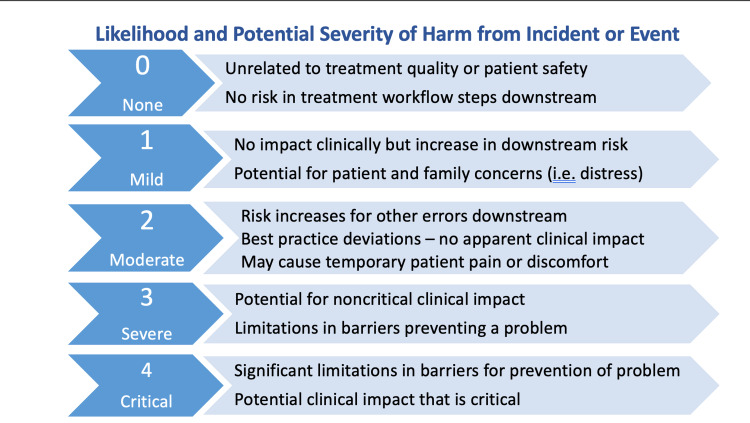
Categories and Likelihood of Potential Incident Severity The ability for staff to score and report the potential severity of a near-miss - an incident or event that could cause harm if it reached a patient, increased staff discussions, learning, and reporting of “good catches” (near misses identified and mitigated early). Adapted from Nyflot et al. [5] for the R-WILS tool. R-WILS: radiation oncology web-based incident learning system

The characterization of incident severity categories was based upon a ‘near miss risk index’ (NMRI) developed by Nyflot et al. [5] and adapted for department-specific reporting (Figure 3):

An R-WILS icon was placed on all department computers for ease of access, anonymized reporting, and concurrent use with the H-ERS throughout the study period. Completed R-WILS incident reports were electronically submitted and auto-uploaded in real-time to a central database specially created for the study. Reported data were aggregated, graphically summarized into custom reports, and prioritized on a daily basis by the chief of medical physics in preparation for analysis by the project committee (Committee) created for this study.

The Committee, chaired by the medical director, consisted of project champions from each functional department area, including dosimetry, medical physics, radiation therapists, nursing, and administration. Committee members performed weekly causal analysis, including evaluation of incident rates, trends, potential severity, and location along the treatment path using custom reports and statistical process control charts. The chief of medical physics and a medical physics designee performed follow-up with individual staff to “close the loop,” whether for positive recognition, additional information, or mitigation. Committee findings and decisions were also shared during weekly team training huddles, giving an opportunity for all “crew” to share additional concerns, insights, positive recognition, as well as recommendations for mitigation.

Measurement strategy

Data collected over the six-month study period included the monthly number and rate of incidents reported; patient treatments per workload; incident severity potential; and step or location of incident origination within the treatment workflow sequence. Incident rates were normalized to the total number of reported incidents in a given month per 1000 patient treatments during the same period and subsequently converted to incidents per 100 fractions (treatments) for industry comparison purposes. Statistical process control charts were used for trending and evaluating significant events over time. Bar graphs and pie charts were used to reflect categorical incident information. Duplicative, incomplete, or retracted reports were excluded from the analysis.

## Results

Reported incidents

**Figure 4:**
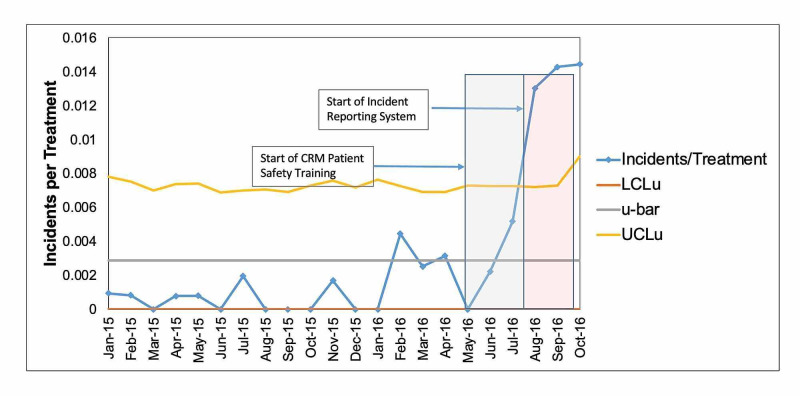
Monthly Trending of Reported Incidents per Phase The number of reported incidents per month normalized to 1,000 patient treatments per month represents the incidents per treatment (fraction). A significant level of reporting (> 3 sigma) was observed during Phase IIB, when the custom incident learning system and project Committee were added to the weekly department CRM training. CRM: Crew Resource Management

During the 22-month study period (Phases I and II), an increasing trend was observed in the mean monthly rate and number of reported incidents, as portrayed in Figure 4. A five-fold increase in the mean number of monthly reported incidents (n = 9.3) was observed during the six-month intervention period compared to the 16-month pre-intervention period (n = 1.8). When the custom reporting system was added during the last three study months, a significant increase (>3 sigma) was observed as evidenced by incidents per treatment exceeding the upper control limit (UCLu). This may be attributable to the staff's improved ability to detect and recognize errors, staff comfort level, and utilizing a discipline-specific incident learning system.

Incident severity classification

**Figure 5:**
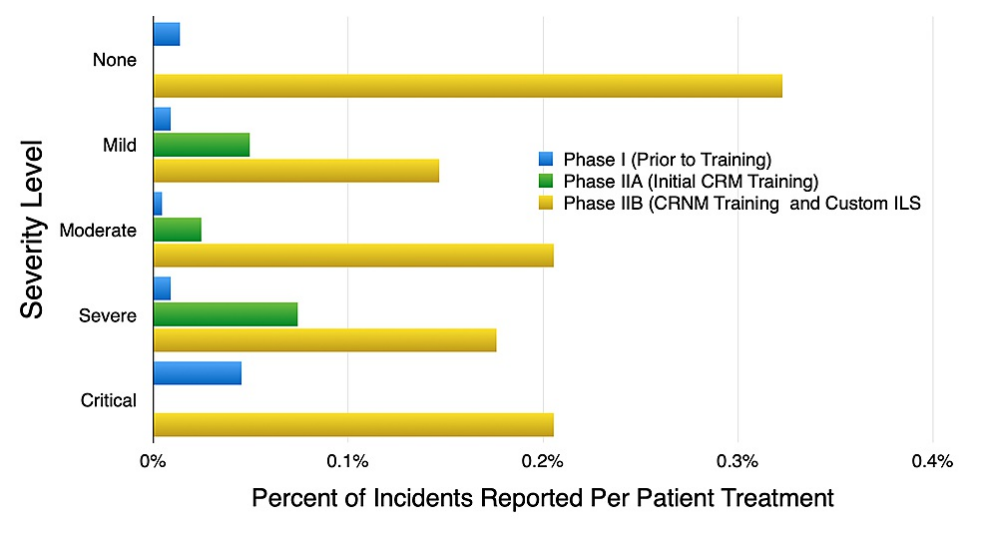
The rate of incidents reported according to the potential for incident severity was greatest when the custom reporting system was added during Phase IIB

The rate of incidents reported per potential severity level was dramatically increased during the last three study months. During this time, the R-WILS was added to weekly CRM training (Phase IIB) as compared to the first three study months (Phase IIA), when CRM training was implemented while staff continued reporting on the hospital system (H-ERS) (Figure 5). The category with the lowest threshold of potential severity was observed as having the greatest number of reported incidents when compared to the other severity categories (n = 14 none vs. n = 8-11 mild to critical) during Phase IIB. This may be attributed to staff's heightened awareness in recognizing near misses and close calls as reportable events, as well as the opportunity to characterize the potential for incident severity when the R-WILS was added during Phase IIB.

Potential severity categories and workflow step

**Figure 6:**
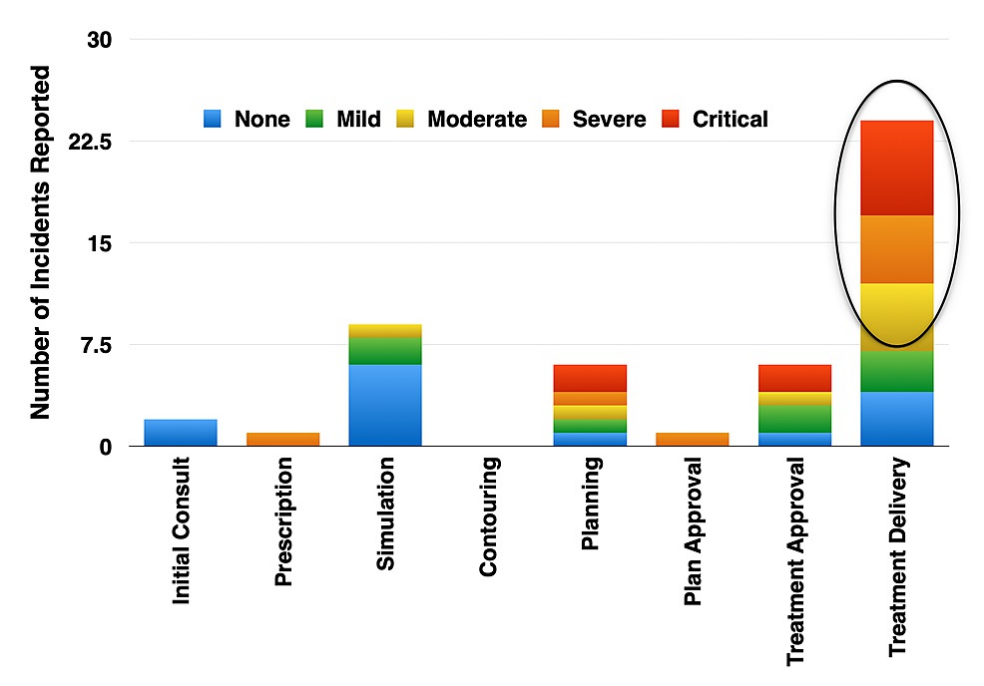
Potential Incident Severity Originating at Steps in the Treatment Workflow The treatment delivery step, which presents the last opportunity in the treatment workflow to mitigate an error, was identified as having the largest number of incidents with the greatest potential for severity.

The reported frequency and severity of incidents detected within a specific workflow step is presented in Figure 6. The largest number of incidents with the greatest potential for (critical) severity were identified at the treatment delivery step, which is the last opportunity to detect and intercept a treatment error. The second-highest number of reported incidents with the greatest potential for (critical) severity were detected at the physics review/approval and treatment planning steps.

The rate of incidents reported according to the potential for incident severity was greatest when the custom reporting system was added during Phase IIB.

## Discussion

As processes in radiation oncology become more complex, the programs that underpin industry best practices for safety and quality must evolve. Incident reporting is a proven tool for directly evaluating changes in a radiation oncology department’s safety program and is an indirect measure of system safety [3]. High-reliability organizations, including commercial aviation, have demonstrated significant reductions in potentially fatal events since adopting structured safety training and mandatory incident reporting programs to optimize team safety performance. Implementing a radiation oncology-specific team training program based upon high-reliability organization (HRO) principles and the three domains of the Joint Commission RPI Model [11] resulted in improvements in the quality and reporting of incidents over a six-month intervention period. Creating and implementing a web-based customized RO incident learning system (R-WILS) after three months of staff training shows the combined intervention is an effective approach for increasing the quality and frequency of departmental incident reporting rates over time. Real-time review and positive acknowledgment of reported incidents in a culture of safety helped promote staff comfort in openly discussing and reporting incidents without fear of retribution. Building the intervention into the department workflow facilitated a systematic approach for (1) staff reporting and learning; (2) real-time opportunities to discuss departmental safety concerns during staff huddles; and (3) increased team collaboration and structured communication concerning incident mitigation throughout the treatment path.

Though published numbers and reporting metrics can vary widely (i.e., per patient, per fraction, etc.), the average rate of reporting (1.39 per 100 fractions) in this study is relatively consistent with that of similar studies [6]. For example, a 46-month low-threshold reporting study that utilized an electronic “condition” reporting program, revealed that a total of 8,504 conditions (average 1 per patient, 185 reports per month, 3.9 per 100 fractions) were reported [6]. The average reporting rate of this study is approximately two to five times the respective rates described by Mutic et al. (0.6 per patient treated) and Clark et al. (0.7 per 100 fractions treated) [6].

Access to a customized R-WILS contributed to the significant increase in RO staff reporting rates, meaningful learning opportunities, and broader staff participation during departmental meetings. The ability to characterize incident severity, location in the care path, and trend detected incidents, provided leadership and staff with a relevant and systematic approach to critically evaluate incidents, improve processes, and “close the loop.” For example, the rate of reporting potential severity in the lowest threshold category (none) was greater than in any of the higher level (mild-critical) categories, which may be a result of training for early identification. This finding may bring into question the significance of increased incident reporting and the early identification of upstream errors as important indicators of system weaknesses or failures [2,4,6,9]. It also presents impactful opportunities for staff education, process improvement, and the proactive prevention of potentially harmful errors - well before they reach a patient.

While incident reporting systems may not include all areas that pose risk for patients, reporting systems, provide an objective means for evaluating actual events occurring during the workflow or to patients themselves [3]. Accordingly, team-based training and participation in the resolution of reported incidents has had a positive impact on the department in areas outside patient safety. Greater clarity and understanding of the entire workflow and patient care path by all staff has resulted in improvements in other non-clinical areas. In fact, staff identified an upward trend in reported incidents with a higher severity potential (critical) originating prior to the treatment delivery step in the workflow during one Committee meeting. Root cause analysis revealed the treatment therapists were inconsistently applying established “sterile cockpit” procedures due to increased workload demands from a recent therapist shortage and an extensive pre-treatment checklist. A “system” strategy created by staff included offloading some “downstream” non-treatment tasks to the appropriate “upstream” functional areas. This enabled the therapists at the treatment console time to focus on essential patient treatment and safety protocols.

The Joint Commission finds that leadership engagement in patient safety and quality initiatives is imperative, as 75% to 80% of all initiatives that require people to change behaviors fail in the absence of leadership’s commitment to change [10]. Leadership and staff participation in weekly multidisciplinary team meetings, training, and “safety rounds” helped establish feelings of openness, respect, participation, and responsibility [9]. Leadership and several staff acknowledged positive changes in areas of team transparency, vigilance, and comfort in discussing and reporting incidents without fear of reprisal. For example, staff were observed openly discussing safety concerns while applying structured communication techniques (i.e., SBAR, etc.) during huddles and in R-WILS reports. In fact, the use of SBAR routinely in the custom incident reporting database - versus the H-ERS - not only improved the quality of reporting, but provided a more systematic, concise, and efficient approach in conveying and managing event information, according to Leadership. The focus of Committee leadership on the “what” rather than the “who” when discussing reported incidents, visibly allayed staff concerns for reporting, appeared to “flatten” professional hierarchies, and foster a culture of non-retribution. Frequent training combined timely feedback and positive staff recognition for effective teamwork, communication, and safety performance from clinical leadership (i.e. medical director, chief of physics, etc.) appeared to reinforce and sustain desired CRM behaviors [16].

The limitations of this study include the voluntary nature of reporting, which is inherently inaccurate. In addition, the planning and execution of this intervention were both time-consuming and resource-intensive. The Department's rapid growth and increasing treatment complexity warrant subtle process changes to the interventions implemented in this study. Accordingly, departmental onboarding and frequent training should be optimized toward team building, change and process management, effective safety communication, and continued customization of the R-WILS. Leadership should continue with efforts towards creating an accountable “upstream” incident mitigation process that empowers staff to immediately (without waiting for a team meeting) evaluate and manage any incident flagged as “critical.” Incentive programs promoting staff engagement and timely feedback at all levels will help promote a culture of safety, hardwire staff safety and reporting behaviors, and accelerate the department on their journey towards high-reliability healthcare.

## Conclusions

Incident learning is recognized as a major cornerstone for sustaining high-quality safety performance in healthcare and other high-reliability industries. This study implemented a dual training and incident learning intervention based upon a high-reliability healthcare framework, resulting in significantly improved incident reporting rates. While increased incident reporting rates may be considered to be a metric for success, rates alone cannot be solely attributable to training and the discipline-specific incident learning system. Both training and the R-WILS provided systematic and structured approaches for detecting, reporting, learning about, and characterizing incidents. Staff comfort in reporting can be attributable to trust in the Leadership's commitment to a culture of safety. This included the Leadership's transparency and visible engagement in routine training, reporting with timely feedback, and incentives designed to hardwire desired team-based safety behaviors. The cyclical nature of the high-reliability health care framework, where staff trust in leadership promotes an increase in team reporting behaviors and subsequent incident learning then translates to ongoing improvement, was foundational to the success of this project. While the implementation of these combined strategies enhanced patient safety, teamwork, and multi-disciplinary collaboration, challenges still remain in effecting the correct incentives for sustainability and metrics that accurately quantify staff safety performance and patient outcomes.
